# Ependymoma Pediatric Brain Tumor Protein Fingerprinting by Integrated Mass Spectrometry Platforms: A Pilot Investigation

**DOI:** 10.3390/cancers12030674

**Published:** 2020-03-13

**Authors:** Diana Valeria Rossetti, Luca Massimi, Claudia Martelli, Federica Vincenzoni, Susanna Di Silvestre, Gianluca Scorpio, Gianpiero Tamburrini, Massimo Caldarelli, Andrea Urbani, Claudia Desiderio

**Affiliations:** 1 Dipartimento di Scienze biotecnologiche di base, cliniche intensivologiche e perioperatorie, Università Cattolica del Sacro Cuore, 00168 Roma, Italy; dianavaleria.rossetti@unicatt.it (D.V.R.); martelli.claudia@gmail.com (C.M.); Federica.vincenzoni@unicatt.it (F.V.); 2 Fondazione Policlinico Universitario A. Gemelli IRCCS, 00168 Roma, Italy; 3 UOC Neurochirurgia Infantile, Dipartimento di scienze dell’invecchiamento, neurologiche, ortopediche e della testa-collo, Fondazione Policlinico Universitario A. Gemelli—IRCCS, Università Cattolica del Sacro Cuore, 00168 Roma, Italy; luca.massimi@unicatt.it (L.M.); suzanneds92@gmail.com (S.D.S.); gianlucashasascorpio@gmail.com (G.S.); gianpiero.tamburrini@unicatt.it (G.T.); massimo.caldarelli@unicatt.it (M.C.); 4 UOC Chimica, Biochimica e Biologia Molecolare Clinica, Fondazione Policlinico Universitario Agostino Gemelli—IRCCS, 00168 Roma, Italy; 5 Istituto di Scienze e Tecnologie Chimiche “Giulio Natta”, Consiglio Nazionale delle Ricerche, 00168 Roma, Italy

**Keywords:** pediatric ependymoma, posterior fossa, proteomics, mass spectrometry

## Abstract

Ependymoma pediatric brain tumor occurs at approximate frequencies of 10–15% in supratentorial and 20–30% in posterior fossa regions. These tumors have an almost selective response to surgery and relative and confirmed resistance to radiotherapy and chemotherapic agents, respectively. Alongside histopathological grading, clinical and treatment evaluation of ependymomas currently consider the tumor localization and the genomic outlined associated molecular subgroups, with the supratentorial and the posterior fossa ependymomas nowadays considered diverse diseases. On these grounds and in trying to better understand the molecular features of these tumors, the present investigation aimed to originally investigate the proteomic profile of pediatric ependymoma tissues of different grade and localization by mass spectrometry platforms to disclose potential distinct protein phenotypes. To this purpose, acid-soluble and acid-insoluble fractions of ependymoma tumor tissues homogenates were analyzed by LC-MS following both the top-down and the shotgun proteomic approaches, respectively, to either investigate the intact proteome or its digested form. The two approaches were complementary in profiling the ependymoma tumor tissues and showed distinguished profiles for supratentorial and posterior fossa ependymomas and for WHO II and III tumor grades. Top-down proteomic analysis revealed statistically significant higher levels of thymosin beta 4, 10 kDa heat shock protein, non-histone chromosomal protein HMG-17, and mono-/uncitrullinated forms ratio of the glial fibrillary acidic protein (GFAP) fragment 388–432 in supratentorial ependymomas—the same GFAP fragment as well as the hemoglobin alpha- and the beta-chain marked grade II with respect to grade III posterior fossa ependymomas. Gene ontology classification of shotgun data of the identified cancer and the non-cancer related proteins disclosed protein elements exclusively marking tumor localization and pathways that were selectively overrepresented. These results, although preliminary, seem consistent with different protein profiles of ependymomas of diverse grade of aggressiveness and brain region development and contributed to enlarging the molecular knowledge of this still enigmatic tumor.

## 1. Introduction

Ependymomas are rare malignances and represent about 10% of all pediatric CNS tumors [1]. More in detail, they account for approximately 10–15% of supratentorial and 20–30% of posterior fossa tumors in children. They can occur at any age; however, posterior cranial fossa ependymomas mainly occur in children (peak of incidence: 4th–6th year of age), often in infants (25–40%) [2]. A DNA methylation profile provided a new ependymoma classification in nine diverse subtypes with different molecular profiles, three per localization in CNS (spinal, posterior fossa, and supratentorial localizations), the group A in posterior fossa, and the REL-associated protein/p65 (RELA) fusion-positive supratentorial ependymomas resulting in worse prognosis, as recently reviewed [1]. Supratentorial and posterior fossa ependymomas are nowadays considered different diseases, and tumor grading should not be considered for clinical evaluation of ependymomas [3].

The main common pitfalls connected with the treatment of these tumors are represented by their almost selective response to surgery with a relative resistance to radiotherapy (RT) and a confirmed resistance to common chemotherapeutic agents. Actually, chemotherapy (CT) is mainly used to postpone RT and/or to reduce the irradiation field, while RT enters treatment protocols to consolidate a gross total surgical resection or in cases of unresectable remnants [4]. The extent of surgical resection remains the main prognostic factor; however, a gross total surgical resection is hardly achievable (50–60% of cases) [2]. This occurs especially in the molecular subgroup A, where the tumor affects young children and typically extends to the cerebello-pontine angle with subsequent encasement of the cranial nerves and the arterial branches of the posterior Willis’ circle [5,6]. Moreover, a significant proportion of children with totally resected posterior fossa ependymomas experience a tumor recurrence [7].

On these grounds, the need to better characterize this tumor to find therapeutic and prognostic markers is particularly felt. Therefore, a proteomic characterization could add important information on ependymomas of different grades and localization.

A review paper in 2014 summarized the results and outlined the perspective of the application of proteomics science to molecular profiling pediatric brain tumors [8]. Although brain tumors are the most frequent solid tumors in the pediatric age, only a few proteomic studies have been published. The two-dimensional difference gel electrophoresis (2D-DIGE) coupled with MALDI-MS was applied to characterize aberrantly expressed proteins in 12 samples of ependymoma tumor tissue and 29 Primitive Neuro-Ectodermal Tumors (PNETs) [9]. While stathmin marked PNETs, ependymomas showed the overexpression of annexin A1 and calcyphosine. In silico proteomics identified pre-B-cell leukemia homebox interacting protein 1 (PBXIP1) overexpressed in ependymomas with respect to a normal brain [10]. A previous study using nano LC high resolution mass spectrometry and the shotgun approach provided the first comprehensive characterization of the proteome of 10 ependymoma tumor tissues of WHO grade II in comparison to other pediatric brain tumors [11]. Based on these data, the same group of authors published the first Pediatric Ependymoma Protein database (PEPD), including 4157 protein groups identified with high confidence [12]. Recently, proteomics was applied to identify the functional domain involved in the binding and the inhibition of polycomb repressive complex 2 (PCR2) function by chromosome X open reading frame 67 (CXorf67), a key factor involved in the oncogenic mechanism of histone 3 K27 hypomethylation and selectively overexpressed in group A posterior fossa ependymomas [13]. To the best of our knowledge, the proteomic profiling of ependymomas tumor tissues of different locations and WHO tumor grades has not been thus far reported. The most recent molecular studies on ependymomas based on their updated classification are based on immunohistochemistry. Immunohistochemistry studies distinguished supratentorial ependymomas in the RELA-fusion-positive and the yes-associated protein 1 (YAP1)-fusion positive subgroups, the Neural cell adhesion molecule L1 (L1CAM) protein marking the first one and showing the most aggressive behavior [14]. CD44 was instead outlined as a biomarker of prognosis of posterior fossa ependymomas as well as being in association with abnormal activation of the phosphatidylinositol 3-kinase (PI3K)/protein kinase B (AKT) pathway (PI3K-Akt pathway) [15]. A study using immunohistochemistry evaluated the expression of Cellular tumor antigen p53 (P53), Proliferation marker protein Ki-67 (Ki67), cyclin D1, Guanine nucleotide-binding protein G(o) subunit alpha (GNAO1), Acid ceramidase (ASAH1), MICOS complex subunit MIC60 (IMMT), and Importin-7 (IPO7) proteins in ependymal tumors in relation to histopathological grade, age, gender, and progression-free and overall survival, in comparison to control tissue to determine possible prognosis biomarkers [16]. Together with the expression of ASAH1 and GNAO1 recognized in ependymal tumors, the results evidenced the underexpression of P53 and the overexpression of Ki67 and cyclin D1 in correlation with tumor grade and relapse and the overexpression of IPO7 and IMMT with survival.

The aim of this pilot study was to characterize the brain tumor tissues of pediatric ependymoma by LC-MS proteomic analysis to originally explore the existence of distinguished protein profiles associated with WHO tumor grade and diverse tumor localization, i.e., supratentorial and posterior fossa, according to the general consensus statements of ependymoma subgroups [3]. In fact, as a major consensus, the clinical management of ependymomas should not be merely based on the histopathological grade, since diverse tumor localizations, i.e., supratentorial and posterior fossa, seem to define distinct diseases, each including specific molecular subgroups identified by genomic profiling [3]. To this purpose, different proteomic analytical approaches were applied to characterize both the intact/undigested and the trypsin digested proteome of 12 ependymoma pediatric brain tumor tissues of diverse grade and localization following a top-down/shotgun integrated platform. Top-down proteomics—analyzing proteins and peptides in their entire state—is a challenging approach to investigating isoforms and post-translational modifications (PTMs), however limited to proteins of low molecular weight, and to studying the naturally occurring peptidome and bioactive protein fragmentome. The bottom-up platform, including shotgun proteomics, is the most classical and commonly used approach in proteomics, applying chemical or enzymatic digestion to proteins before LC-MS analysis. By this strategy, large-scale protein identification, including proteins of high molecular mass, is allowed, resulting in a large data set suitable for gene ontology analysis, pathways classification, and protein–protein interaction evaluation. The two approaches, although providing different information, were complementary with each other and established an integrated analytical platform for a preliminary proteomic characterization of pediatric ependymoma tumor tissue of diverse grade and localization, which was, to the best of our knowledge, not previously investigated.

## 2. Results

After ependymoma (EP) tissue homogenization, the resulting acid-soluble fraction was processed by LC-MS analysis following the top-down proteomics approach, while the acid insoluble pellet was analyzed by the shotgun strategy. The combination of the overall resulting data provided a comprehensive characterization of EP tumor tissue proteome matching the challenging features and balancing the pitfalls of both analytical approaches. Top-down proteomics typically profiles small proteins and peptides, including the challenging identification of naturally occurring bioactive protein fragments, the so-called “cryptic peptides” [17] of relevance in clinical proteomic studies. This approach, by manual inspection of the MS/MS spectra and de novo sequencing, can precisely identify and localize PTMs along the sequence, which are interesting to investigate with respect to the diverse tumor locations and the grade of aggressiveness. Furthermore, the most selective analysis of the undigested proteome supports the identification of minor proteins and peptides generally difficult to disclose when large data sets are produced, such as by bottom-up/shotgun proteomics. On the other hand, shotgun proteomics—analyzing proteins mixtures after enzymatic digestion—is the approach of excellence for achieving a large number of identification data sets, including high molecular weight proteins, and for investigating their functional interactions, gene ontology (GO) classification and molecular pathways distribution by bioinformatics tools. The obtained results are illustrated herein based on the proteomic approach used. All data are then discussed in a separate paragraph by integrating the results obtained by the two proteomic approaches applied.

### 2.1. Top-Down Proteomic Analysis

Table 1 reports the list of proteins and peptides identified in EP specimens (data in Materials and Methods section) following the top-down proteomic approach. Proteins/peptides were characterized through full MS spectra deconvolution, providing monoisotopic and average molecular mass values of the proteins, and by manual inspection of the MS/MS fragmentation spectra for amino acid sequencing and PTMs identification and localization.

Protein identification was further confirmed by comparison of the experimental/theoretical MS/MS fragmentation data through Prosight light free tool for top-down proteomics (top-down tandem MS identification data of the proteins listed in Table 1 are reported in Table S1).

As can be observed in Table 1, according to the features of the applied approach, the identified elements enclosed small proteins with molecular mass values within 16 kDa and numerous peptides mostly generated from naturally occurring fragmentation of major proteins; the numerous peptide fragments of vimentin and glial fibrillary acidic protein (GFAP) are some examples. Several proteins and peptides PTMs were identified, mainly including N-terminal acetylation, methylation, citrullination, and deamidation. High resolution mass spectrometry allowed us to characterize isobaric peptides corresponding either to different fragments of the same protein or to diverse proteoforms of the same peptide carrying distinguished PTMs, sometimes producing a shift of chromatographic retention time.

**Figure 1:**
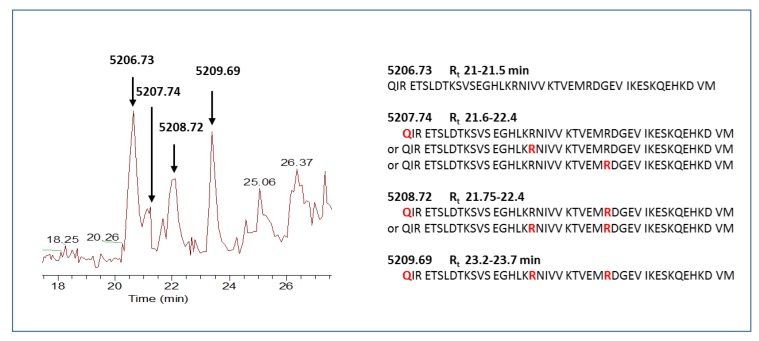
Enlarged view of the LC-Orbitrap Elite MS eXtracted Ion Current (XIC) plot of the glial fibrillary acidic protein (GFAP) fragment 388–432 5206.73 Da, ([M+H]^+^) and its mono- and poly-citrullinated forms. The relative amino acid sequences with the position of the PTMs (Q_deamidation_, R_citrullination_, in red) are also reported.

Different GFAP and vimentin peptide fragments were found in either their unchanged or mono- or poly-citrullinated forms with different localization of the PTM (Table 1 and Table S1). As an example, Figure 1 reports the eXtracted Ion Current (XIC) plot of the GFAP fragment peptide 388–432 in which the uncitrullinated (5206.73 Da) and the diverse deamidated/citrullinated forms producing a delta mass increase can be distinguished in the chromatogram.

**Figure 2:**
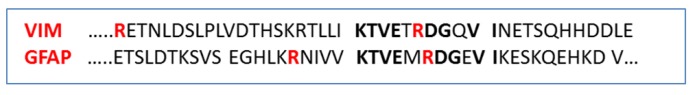
Amino acids sequence traits of GFAP and vimentin (VIM) peptide fragments where citrullation PTMs occur.

It was further interesting to observe that the peptide fragments of both GFAP and vimentin showing citrullination often enclosed the PTM in very similar sequence traits, namely on the arginine residue (in red) inside the sequence trait -KTVEXRDGXVI-. (Figure 2).

**Figure 3:**
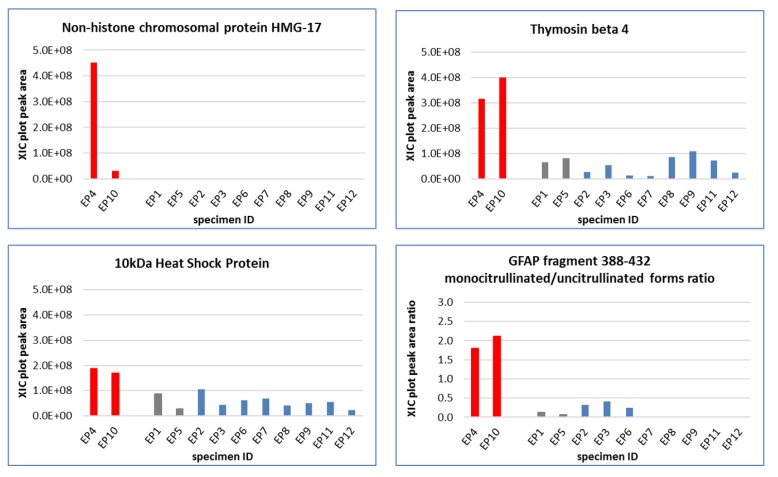
Proteins with statistically significant level variations between supratentorial (ST) and posterior fossa (PF)-EPs.

**Figure 4:**
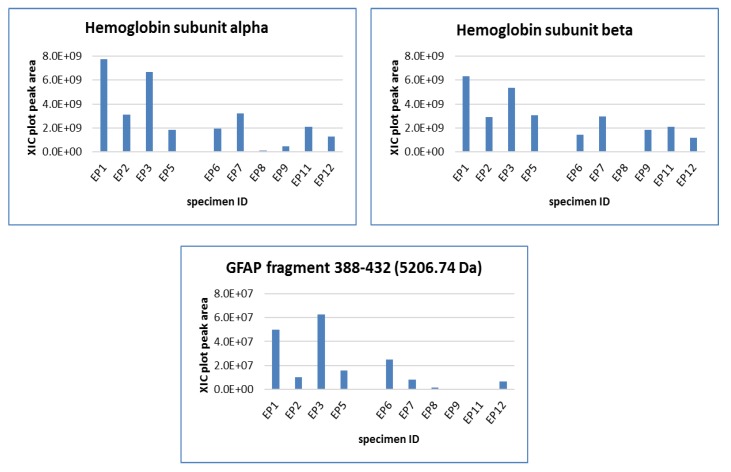
Proteins with statistically significant variation between WHO grade II (EP1–3, EP5) and grade III (EP6–9, EP11, EP12) PF ependymomas.

Relative quantitation of the protein and the peptides in Table 1 showed few elements exhibiting a statistically significant variation between the diverse tumor localization, i.e., posterior fossa (PF) and supratentorial (ST) ependymomas (EPs) (Figure 3), and grade II and grade III PF-EPs (Figure 4). Non-histone chromosomal protein HMG-17 (HMGN2) was almost undetectable in PF-Eps, while quantifiable levels were recognized in ST-EPs. Its variation was highly statistically significant (*p* < 0.01), therefore marking the ST-EP subgroup. Relevant variations between PF and ST-EPs were also found for Thymosin beta-4 (TMSB4X) peptide and 10 kDa heat shock protein, mitochondrial (HSPE1), both exhibiting higher levels in ST-EPs.

Interestingly, the ratio of the monocitrullinated/uncitrullinated form of the fragment 388-432 of GFAP (5207.74/5206.74, [M+H]^+^ monoisotopic masses) showed statistically significant and different results (*p* value < 0.0001) based on tumor localization (Figure 3). While the two single proteoforms did not exhibit significant alterations in relation to tumor localization, their peak area ratio (citrullinated/uncitrullinated form) showed instead a strong increase in ST-EPs (*p* value 4.99744 × 10^−8^), corresponding to average values of 1.96 ± 0.22 with respect to 0.12 ± 0.16 of PF-EPs.

Other proteins showed statistically significant higher levels in ST-EPs with respect to PF-EPs, although with higher *p* values (*p* < 0.05), namely, ubiquitin (UBC), superoxide dismutase (Cu-Zn) (SOD1), parathymosin (PTMS), and AcylCoA binding protein natural variant M→V (DBI) (Figure S1). The analysis of a higher number of ST-EPs could clarify their significance in marking ST-EPs.

The PF-EPs subgroup data were also analyzed to compare the protein profiles associated with different histopathological classification. Although the WHO tumor grade classification is weakened nowadays for ependymomas [3], few proteins intriguingly exhibited a statistically significant variation between grade II (EP 1–3, 5) and grade III (EP 6–9, 11, 12) PF-EPs, all exhibiting increased levels in the tumor tissue of lower grade, namely, the alpha (HBA1) (*p* = 0.03) and the beta (HBB) (*p* = 0.01) hemoglobin subunits and the fragment 388–432 of GFAP (*p* = 0.04) (Figure 4). No protein elements distinguished lateral (EP1 and EP5) from median line PF-EPs.

Although without statistical significance in discriminating tumor localization or tumor grade, the top-down proteomic analysis characterized in ependymoma tissue other proteins and peptides that were already highlighted in our previous studies on other pediatric cerebral tumors in posterior cranial fossa [19,23]. They include ubiquitin and its C-terminal Gly-Gly dipeptide truncated form (Ubiquitin des-GG) —marking the most aggressive medulloblastoma—other fragments of GFAP and vimentin proteins, the bioactive C-terminal fragments of alpha-1-antichymotrypsin and alpha-1-antitrypsin, the C-terminal peptide 375–418 of the latter with reported immunomodulatory activity [24], which would require further investigation to understand the actual biological role in the context of brain tumors. The AcylCoA binding protein was co-characterized in the present investigation with its natural variant M→V, already cited above for its overexpression in the ST-EPs subgroup. S100B and histones H4 and H2A were characterized in their acetylated (N-terminal) form, the latter also identified in their methylated or diacetylated forms. Mitochondrial proteins such as ATP synthase subunit e, ATP synthase coupling factor 6 and cytochrome C oxidase subunit 6B1 did not show interesting quantitative alterations between grade II and grade III EPs or ST- and PF-EPs as either the alpha defensins 1, 2, and 3 involved in inflammation.

### 2.2. Shot-Gun Proteomic Analysis

Tandem MS shotgun proteomic data of ependymoma tumor tissue resulting from the LC-Orbitrap Elite MS analyses were elaborated by the Proteome Discoverer software for protein identification. The resulting data were further filtered for high confidence peptide identification, peptide rank 1, and protein identification by at least two peptides of the minimum of nine amino acid residues according to the Human Proteome Project Mass Spectrometry Data Interpretation Guidelines [25]. Table S2 reports the shotgun protein identification data per analyzed specimen as a result of triplicate analysis per sample.

Venn diagram analysis of the proteins identified per tumor specimen by shot-gun LC-MS analysis identified an overall number of 11,313 unique protein elements in the 12 analyzed ependymoma tumor specimens. This number reduced to 3182 elements after filtering the results, as reported above.

As for top-down results and following the recent guidelines for classification of pediatric ependimoma [26,27], the difference in the proteomic profiles of PF- and ST-EPs was at first investigated by this proteomic approach. The list of the Uniprot accession of the proteins identified per sample is reported in Table S3A. At first, the Venn diagram construction of the protein elements identified in all analyzed samples (graphical representation not available, full data are in Table S3B) allowed us to distinguish proteins exclusively identified in the ST- (EP 4, 10) or the PF-EP (EP 1, 2, 3, 5–9, 11, 12) subgroups. PF-EPs distinguished from ST-EPs for the exclusive detection of two proteins, specifically the heat shock-related 70 kDa protein 2 (HSPA2) and the ribosome-binding protein 1 (RRBP1). Considering the very poor protein profile of the EP2 specimen, its exclusion from the PF-EPs subgroup led to the identification of a third protein distinguishing all the remaining PF-EPs from the ST-EPs, namely, the monocyte differentiation antigen CD14.

On the other hand, ST ependymomas, i.e., EP4 and EP10 specimens, distinguished from PF-EPs for eight exclusive shared protein elements, namely, nuclear fragile X mental retardation-interacting protein 2 (NUFIP2), lipoamide acyltransferase component of branched-chain alpha-keto acid dehydrogenase complex, mitochondrial (DBT), signal transducing adapter molecule 1 (STAM), L-xylulose reductase (DCXR), allograft inflammatory factor 1-like (AIF1L), transcription elongation factor A protein-like 5 (TCEAL5), Erbin (ERBIN), and SUN domain-containing protein 2 (SUN2). Looking at potential differences among PF-EPs of diverse locations inside the IV ventricle, i.e., the lateral/pontine angle samples (EP1 and EP5) with respect to the median line (all other PF samples), only one element—the HLA class I histocompatibility antigen A alpha chain protein (HLA-A)—distinguished the median line from the later/pontin angle localization.

Together with looking to exclusive protein elements marking one or another EP subgroup, the gene ontology (GO) analysis allowed us to investigate and compare their pathways overrepresentation with the PANTHER tool (detailed reference in Data Analysis paragraph 4.5.). At first, ST and PF-EPs underwent sample grouping analysis to outline the list of proteins that commonly identified the subgroups. As resulting from Venn diagram elaboration, ST-EPs shared 922 unique protein elements out of the 1671 identified, while the PF-EPs showed a number of 295 out of the 3023 characterized.

**Figure 5:**
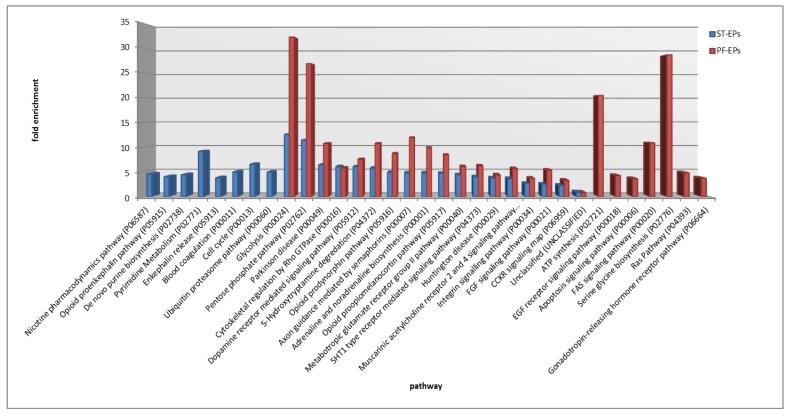
Pathways overrepresentation analysis of the common proteins of ST-EPs and PF-EPs subgroups.

The pathway overrepresentation analysis with the PANTHER tool of these common lists showed 17 pathways overrepresented with statistical significance [false discovery rate (FDR) *p* < 0.05, with respect to human genome] in both subgroups, while eight and seven pathways were exclusively overrepresented in ST- and PF-EPs, respectively (Figure 5). As can be observed in the graph, some of the pathways commonly overrepresented in ST- and PF-EPs showed interesting differences, particularly evident for the glycolysis and the pentose phosphate metabolic pathways, with 5-hydroxytryptamine degredation, opioid prodynorphin pathway, axon guidance mediated by semaphorins, adrenaline and noradrenaline biosynthesis and opioid proopiomelanocortin pathways all exhibiting in PF a noticeably higher fold enrichment value with respect to the ST subgroup.

The same lists were therefore filtered for cancer related proteins by matching the protein elements identified with the cancer related genes list reported in the Human Protein Atlas (http://www.proteinatlas.org) (detailed reference in Data Analysis paragraph 4.5.) and classified by gene ontology analysis by the PANTHER tool.

ST-EPs shared 191 cancer related proteins, while PF-EPs shared 83, with 80 of them common to both subgroups and therefore representative of ependymoma tumor independently from localization or tumor grade.

**Figure 6:**
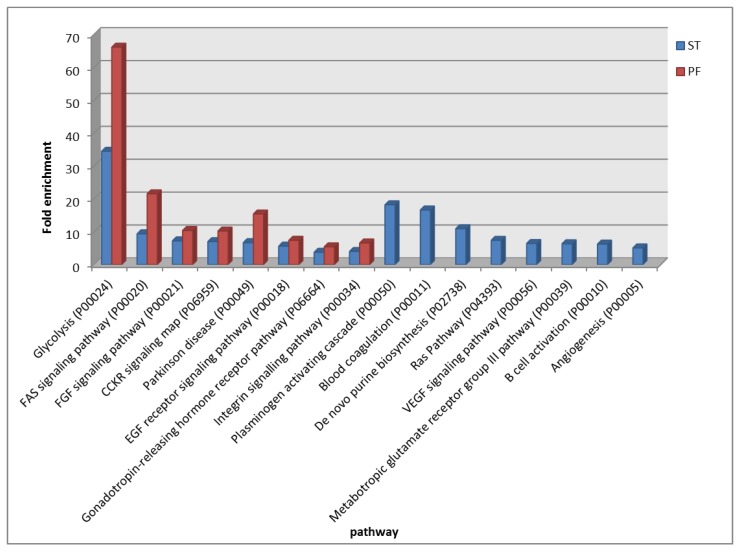
Pathways overrepresentation analysis of the cancer related protein list common to each ST- and PF-EPs subgroup.

Figure 6 illustrates the comparison of the pathways with statistically significant (FDR *p* < 0.05, with respect to human genome) overrepresentation in ST- and PF-EPs, exclusively considering the cancer related proteins. Differently from considering the whole proteins identified, this analysis showed some dissimilarities in the pattern of overrepresented pathways assigned to the ST and the PF subgroups; however, glycolysis (P00024), Fibroblast growth factor (FGF) (P00021) and Cholecystokinin receptor (CCKR) (P06959) signaling, Parkinson’s disease (P00049), and integrin signaling (P00034) pathways were confirmed as overrepresented in both tumor localization subgroups, and blood coagulation and de novo purine biosynthesis were confirmed as exclusively overrepresented in ST-EPs. In this overrepresentation analysis, it is noteworthy to underline the presence of exclusive pathways only for ST-EPs, namely, plasminogen activating cascade (P00050), blood coagulation (P00011), de novo purine biosynthesis (P02738), Ras pathway (P04393), Vascular endothelial growth factor (VEGF) signaling pathway (P00056), metabotropic glutamate receptor group III pathway (P00039), B cell activation (P00010), and angiogenesis (P00005) pathways. The pathways overrepresented in both subgroups generally showed higher values of fold enrichment in PF-EPs, particularly relevant for the glycolysis pathway (P00024), similarly to previous findings, and FAS signaling (P00020).

**Figure 7:**
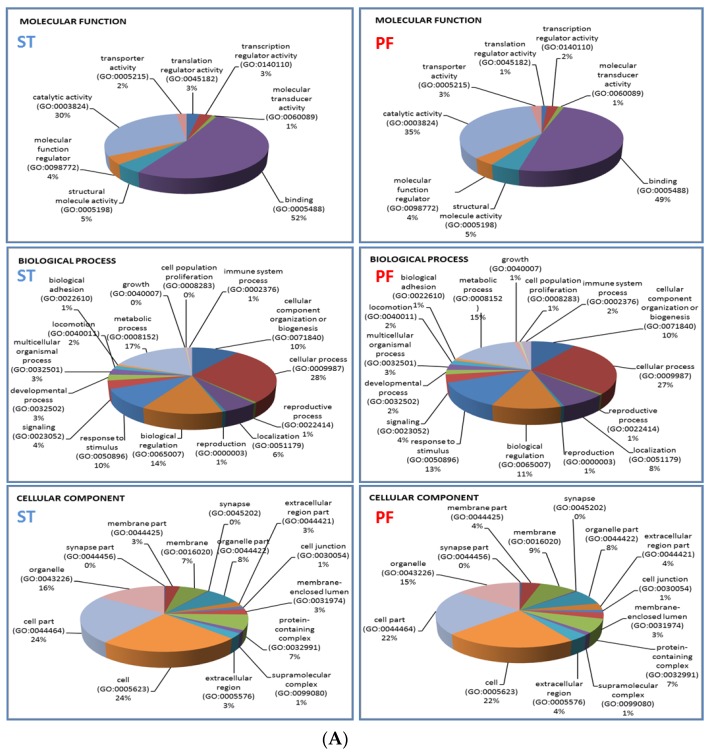

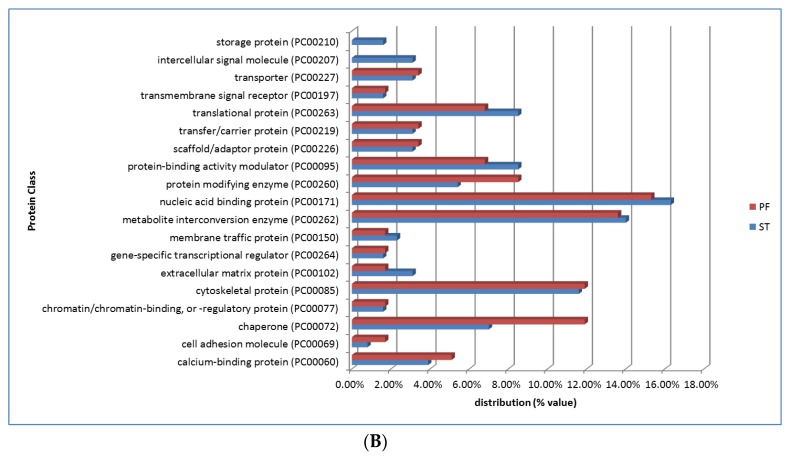
(**A**) Molecular function, biological process, and cellular component gene ontology (GO) classification of the 191 and the 83 cancer related protein lists common to ST- and PF-EP specimens, respectively. (**B**) Protein class GO classification of the 191 and the 83 cancer related protein lists common to ST- and PF-EP specimens, respectively.

GO classification of the ST and the PF-EPs cancer related protein lists based on molecular function, biological process, and cellular component did not evidence valuable differences between the subgroups (Figure 7A), which was also in accordance with the majority of PF-EPs protein elements in common with ST-EPs. Binding and catalytic activities were prevalent in classification of molecular function, while cellular and metabolic processes followed by biological regulation and response to stimulus were predominant inside the biological process. Cell part, cell, and organelle were major in cellular component classification; however, membrane and extracellular components were also classified.

Relative to protein classes, ST-EPs showed, although with a low percentage value of distribution, the exclusive classification of storage protein and intracellular signal molecules protein classes (Figure 7B), the first including ferritin light and heavy chain, and the second including fibrinogen beta and gamma chains, ephrin-B1, and osteopontin. Besides, it is noteworthy to underline the different distribution of the chaperone protein class, corresponding to 7.0% and 11.9% for ST- and PF-EPs, respectively.

Although the WHO classification is currently controversial for ependymoma brain tumor [3], it was interesting to compare the protein profiles of grade II and grade III EPs inside the PF subgroup, enclosing the highest number of analyzed specimens to disclose eventual variations associated with tumor aggressiveness.

It is noteworthy to underline that the number of overall identified protein elements was higher in grade III (EP6–9, 11, 12) than in grade II (EP1, 2, 3, and 5) PF-EPs, namely, 2926 with respect to 1410. Venn diagram grouping based on tumor grade resulted in 570 and 323 protein elements shared by grade III and grade II specimens, respectively (data in Table S3C,D, respectively). In particular, out of them, 295 proteins were in common, while 28 and 275 elements were exclusive of grade III and grade II PF-EPs, respectively. These lists underwent PANTHER pathway classification and overrepresentation analysis and were then compared (data in Table S3E).

**Figure 8:**
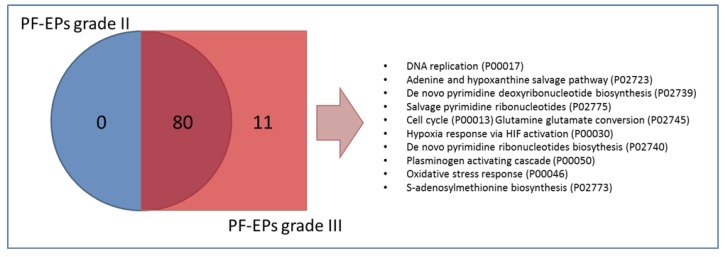
Venn diagram representation of the pathways classified in grade II and grade III PF-EPs. The arrows indicate the description of the 11 pathways exclusively classified in grade III PF-EPs.

Resulting from pathways classification and Venn diagram grouping (Figure 8), from the total number of 91 pathways classified, 11 pathways were exclusively catalogued in grade III PF-EPs, namely, DNA replication (P00017), adenine and hypoxanthine salvage pathway (P02723), de novo pyrimidine deoxyribonucleotide biosynthesis (P02739,) salvage pyrimidine ribonucleotides (P02775), cell cycle (P00013), glutamine glutamate conversion (P02745), hypoxia response via Hypoxia-inducible factor (HIF) activation (P00030), de novo pyrimidine ribonucleotides biosynthesis (P02740), plasminogen activating cascade (P00050), oxidative stress response (P00046), and S-adenosylmethionine biosynthesis (P02773), disclosing potential molecular mechanisms to be further investigated. No pathways exclusively classified in grade II PF-EPs were recognized.

**Figure 9:**
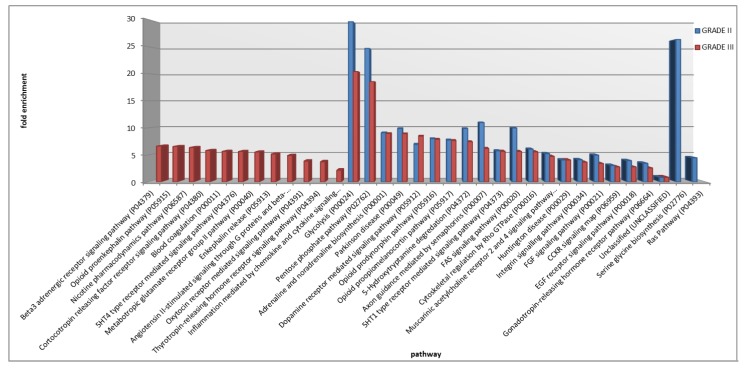
Pathways overrepresentation analysis of WHO grade II and grade III PF-EPs common protein lists.

Figure 9 shows the list of PANTHER pathways for comparison with statistically significant overrepresentation (FDR *p* ≤ 0.05, with respect to human genome) in grade II and grade III PF-EPs with fold enrichment values in *y*-axis. Two pathways were exclusively overrepresented in grade II PF-EPs, namely, the serine glycine biosynthesis (P02776) and the Ras (P04393) pathways, while 12 pathways were exclusively overrepresented in grade III PF-EPs, i.e., ortocotropin releasing factor receptor signaling pathway (P04380), thyrotropin-releasing hormone receptor signaling pathway (P04394), nicotine pharmacodynamics pathway (P06587), oxytocin receptor mediated signaling pathway (P04391), opioid proenkephalin pathway (P05915), metabotropic glutamate receptor group II pathway (P00040), inflammation mediated by chemokine and cytokine signaling pathway (P00031), enkephalin release (P05913), beta3 adrenergic receptor signaling pathway (P04379), blood coagulation (P00011), angiotensin II-stimulated signaling through G proteins and beta-arrestin (P05911), and 5HT4 type receptor mediated signaling pathway (P04376).

As previously performed for ST- and PF-EPs, the protein lists associated with grade II and grade III PF-EPs were filtered for cancer related proteins. Out of the 323 proteins common to grade II PF-EPs, 91 were classified as cancer related proteins, while in grade III specimens, they were 129 out of the total 570. Their grouping by Venn diagram elaboration identified eight and 43 proteins as selective of grade II and grade III EPs, respectively, and 83 commonly represented independently from tumor grade. In accordance with previous findings, these 83 cancer related proteins confirmed the overrepresentation of the FAS and the FGF signaling, the CCKR signaling map, the Parkinson’s disease, the EGF receptor signaling, the integrin signaling, and the gonadotropin-releasing hormone receptor pathways.

The following attention was focused on the eight and the 43 cancer related proteins exclusive of the lower and the higher tumor grades, respectively, performing their pathway overrepresentation analysis. In grade III PF-EPs, statistically significant overrepresentation was found for de novo pyrimidine ribonucleotides biosynthesis, de novo pyrimidine deoxyribonucleotide biosynthesis, plasminogen activating cascade, glycolysis, de novo purine biosynthesis, and blood coagulation pathways.

**Figure 10:**
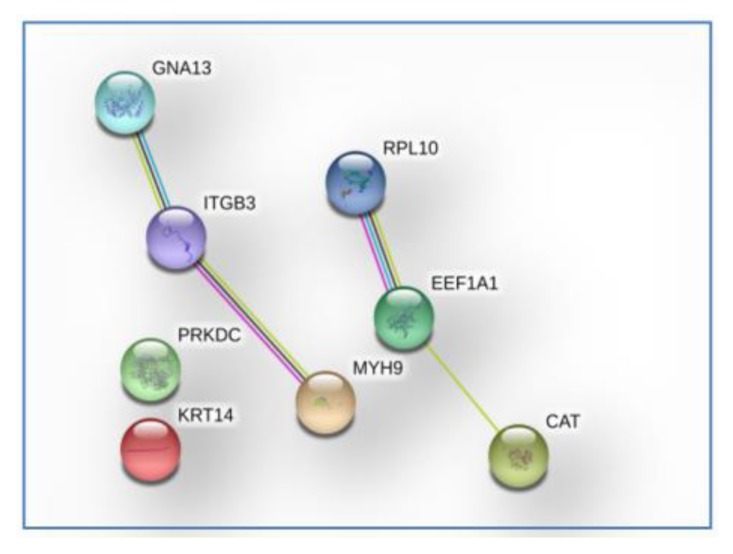
Protein–protein association network (medium confidence score 0.400) of the eight cancer related proteins selectively identified in grade II PF-EPs.

The identification of only eight proteins distinguishing grade II from grade III PF-EPs, namely, guanine nucleotide-binding protein subunit alpha-13 (GNA13), elongation factor 1-alpha 1 (EEF1A1), DNA-dependent protein kinase catalytic subunit (PRKDC), keratin, type I cytoskeletal 14 (KRT14), catalase (CAT), integrin beta-3 (ITGB3), myosin-9 (MYH9), and 60S ribosomal protein L10 (RPL10), generated no results of pathways overrepresentation. Their STRING tool (detailed reference reported in Data Analysis paragraph 4.5) analysis in medium confidence evidenced a network of functional interaction for some of them (Figure 10), specifically between MYH9/GNA13/ITGB3 and RPL10/EEF1A1/CAT, the first group involved, following the KEGG tool (detailed reference reported in Data Analysis paragraph 4.5) annotation in regulation of actin cytoskeleton and platelet activation pathways. These two pathways were not classified inside the 43 cancer related proteins exclusive of grade III PF-EPs.

## 3. Discussion

The present results show the successful integration of two different proteomic approaches for profiling tumor tissues of ependymoma pediatric brain tumors of diverse localizations and histopathological grades. Although few specimens of ST tumor localization were available to be analyzed, it was interesting to compare their proteomic profile with PF tumor tissues as preliminary data. Nonetheless, inside the PF-EP subgroup, the differences between WHO grade II and grade III specimens were also investigated. Both tumor grade and localization resulted in distinguished proteomic profiles either considering the total proteins identified or exclusively the cancer related proteins. Both sets of information are relevant and provide different data, one exclusively concerning known cancer pathways and the other possibly disclosing new correlations, protein elements, or molecular mechanisms potentially involved in ependymoma onset and progression.

**Figure 11:**
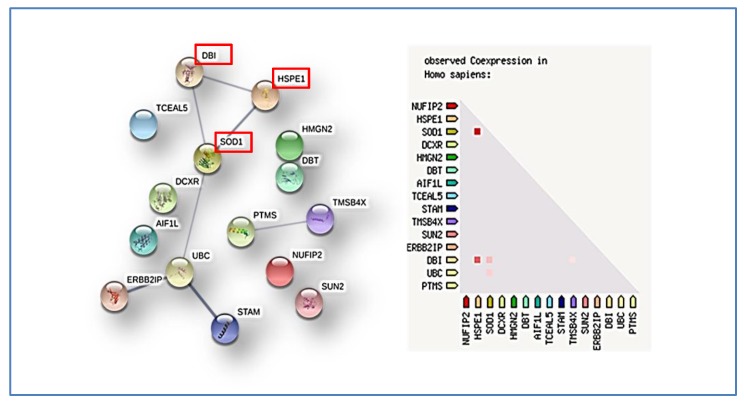
Protein–protein association network (medium confidence 0.400) (left panel) and co-expression (right panel) of the proteins elements distinguishing ST- from PF-EPs, resulting from integrated top-down/shotgun proteomic analysis. Cancer related proteins are marked in red.

The differences in the identified protein elements exclusively detected in one or another subgroup, the GO classification, and the overrepresentation analysis were therefore evaluated. Particularly, considering the combination of the data obtained by both proteomic approaches, i.e., the proteins with statistically significant label free quantitation differences in top-down analysis and the exclusive proteins detected by shot-gun analysis in the two subgroups, a total of 15 protein elements were distinguished ST- from PF-EPs. Their analysis by STRING tool disclosed a network of functional interaction and co-expression for some of them, as represented in Figure 11.

The 10 kDa heat shock protein (HSPE1), Erbin (ERBIN), signal transducing adapter molecule 1 (STAM), superoxide dismutase (Cu-Zn) (SOD1), ubiquitin-C (UBC), and Acyl-CoA-binding protein (DBI) are functional interacting nodes in the network, as well as parathymosin (PTMS) and thymosin beta 4 (TMSB4X). It is interesting to underline that DBI, HSP1, and SOD1 are cancer related proteins, as reported in the Human Atlas database. The KEGG tool classifies UBC and DBI inside the same pathway, i.e., the Peroxisome proliferator-activated receptor (PPAR) signaling pathway, while DBT and DCXR are inside the metabolic pathway. STAM was classified in endocytosis and Janus kinase/signal transducers and activators of transcription (JAK-STAT) signaling, while ERBIN was classified in the NOD-like receptor signaling pathways. SOD1 was shown by KEGG to be involved in diverse pathways, i.e., longevity regulating pathway, amyotrophic lateral sclerosis (ALS), prion diseases, peroxisome, and Huntington’s disease. ERBIN interacted with the oncogenic ERBB2 tyrosine kinase receptor and was reported as a promoter of tumor growth by ERBB2 [28] and as a tumor suppressor by negatively regulating both Akt and RAF/MEK/ERK signaling [29]. Non-histone chromosomal protein HMG-17 (HMGN2), thymosin beta-4 (TMSB4X), 10 kDa heat shock protein (HSPE1), and the ratio between citrullinated/uncitrullinated GFAP fragment 388–432 showed significantly higher levels in ST-EPs, therefore marking the ST tumor location. It could further be supposed that the two analyzed ST-EP samples belong to diverse ST phenotype, namely, the RELA-fusion-positive and the YAP1-fusion-positive phenotypes, due to the detection of the L1CAM and the YAP1 protein [27], respectively, in EP10 and EP4 specimens. The RELA-fusion-positive phenotype of EP10 would also be in accordance with its histopathological classification as WHO grade III.

Parathymosin (PTMS) and thymosin beta 4 (TMSB4X) interacting nodes belong to the thymosin family and are involved in binding and polymerization of actin and therefore in cell motility, proliferation, and inflammation processes [30,31]. Thymosin beta 4 and thymosin beta 10 peptides as well as their related C-terminal truncated forms identified in ependymoma tissue by top-down approach were already characterized by our group in other tissues of PF pediatric brain tumors, marking the medulloblastoma aggressive histotype [19]. Thymosin beta 4 exerts various functions in the organism, since it is involved in cell differentiation and migration as well as morphogenesis and organogenesis and acts in angiogenesis, tissue repair, tumor growth, and development of metastasis processes. The role of thymosin in cancer was previously reviewed [32]. In tumor progression, it inhibits apoptosis and allows the escape of immune surveillance. Thymosin beta 4 overexpression in the ST-EPs could be therefore in agreement with the worst prognosis of this ependymoma subgroup.

Inside the list of proteins identified by top-down approach, a separate discussion was devoted to the diverse peptide fragments of GFAP and vimentin and of their citrullinated forms naturally occurring in the ependymoma tissue intact proteome. GFAP and vimentin are important components of the intermediate filaments in mature astrocytes of the central nervous system with a role, as members of the cytoskeleton protein family, in the processes of cell motility and stability. High levels of GFAP can correlate with trauma, inflammatory, and tumor pathologies [33]. The vimentin has a role in the maintenance of cell integrity and resistance to stress. Its overexpression in tumors correlates with disease progression, presence of metastases, and poor prognosis [34].

The citrullination PTM of GFAP and vimentin, reported as due to the action of the Protein-arginine deiminase type-2 (PAD2) enzyme in brain tissue [35,36], produces the loss of charge of the arginine residue, resulting in longer chromatographic retention time (see Figure 1 as an example) of the modified peptide due to its increased hydrophobicity. This PTM alters the folding and the biological function of the protein, and it is reportedly involved in the pathogenesis of autoimmune diseases, inflammation, tumor biology, and progression [37,38]. To the best of our knowledge, the detection of citrullinated GFAP and vimentin peptides has not been reported in pediatric brain tumor tissues. Moreover, it is interesting that the ratio of the unmodified/monocitrullinated forms of the GFAP fragment 388–432 showed a difference in ST- and PF-EPs, exhibiting a higher value in the ST subgroup.

On the other side, PF-EPs distinguish from ST-EPs for the detection of the following proteins, namely, heat shock-related 70 kDa protein 2 (HSPA2), ribosome-binding protein 1 (RRBP1), and monocyte differentiation antigen CD14 (CD14) proteins, resulting from shotgun data. These proteins are involved in different pathways; however, it was interesting to observe that groups of two proteins were classified by KEGG inside the same pathway, namely, HSPA2 and CD14, both cancer related proteins, inside the MAPK signaling pathway and HSPA2 with RRBP1 inside the protein processing in the endoplasmic reticulum pathway, which could be interesting to further investigate in future studies.

The complete list of proteins identified in ST- and PF-EPs samples was submitted to PANTHER pathways overrepresentation analysis. In addition to sharing overrepresented pathways, the two subgroups showed the overrepresentation of exclusive pathways, as already discussed in the Results section. Particularly, PF-EPs exhibited a high fold increased representation of the pathways of ATP synthesis involved in metabolism and serine glycine biosynthesis, driving oncogenesis when hyperactivated [39], and FAS signaling, all not overrepresented in ST. On the other hand, ST-EPs exhibited the exclusive overrepresentation of pathways involved in purine and pyrimidine metabolism, cell cycle, and opioid peptides pathways. Specifically, in relation to the latter, the expression of both opioid receptors and their ligands in tumor cells suggested their role in tumor progression [40]. Additionally, non-classical opioid peptides, the hemorphins, were reported in our previous paper as potential biomarkers of prognosis in posterior fossa pediatric brain tumor cerebrospinal fluid [41].

A different pathway overrepresentation analysis was instead obtained when considering only the list of cancer related proteins identified in ST and PF-EPs as a result of different protein data clustering. However, glycolysis, FGF signaling, CCKR signaling, Parkinson’s disease, and integrin signaling pathways were confirmed as commonly overrepresented in both the subgroups and therefore were overrepresented in ependymoma tumor independently from localization and tumor grade. In this data elaboration, FAS signaling was not exclusive of PF-EPs; nevertheless, the pathway fold increase was noticeably higher in PF-EPs than in the ST- subgroup. Generally, PF-EPs did not result in the overrepresentation of exclusive pathways associated with their own list of cancer related proteins, while ST-EPs selectively showed blood coagulation, VEGF signaling, RAS, metabotropic glutamate receptor group III, B cell activation, and angiogenesis pathways overrepresentation.

The GO classification of the cancer related proteins in ST and PF-EPs revealed differences in the protein class where ST-EPs showed the exclusive classification of storage protein and intracellular signal molecules, although with low percentage value of distribution.

Overall, either considering the cancer or the non-cancer related proteins identified in ST and PF ependymomas, the diverse tumor localization seemed to be associated with distinct proteomic profiles.

Although the WHO classification is controversial for ependymoma following recent literature [3], we recognized interesting differences in the proteomic profiles of grade II and grade III PF tumor specimens analyzed by applying either the shot-gun or the top-down proteomic approaches. The KEGG tool pathway classification of the 275 proteins exclusive of grade III PFs showed the following pathways as including the highest number of proteins, namely, metabolic (42 elements)—mainly involving glucose and purine/pyrimidine metabolism according to metabolic reprogramming in cancer [42]—ribosome (14 elements), regulation of actin cytoskeleton (14 elements), focal adhesion (nine elements), and MAPK signaling (eight elements, including CD14 protein) pathways.

The comparative evaluation of pathway classification and overrepresentation analysis in grade II and grade III PF-EPs revealed interesting differences. Focusing on the most aggressive histotype, grade III PF-EPs distinguished from grade II PF-EPs for the classification of 11 pathways involving de novo pyrimidine deoxyribonucleotide biosynthesis and ribonucleotides salvage, response to oxidative stress and hypoxia, and cell cycle, according to their higher aggressiveness and proliferation capability.

The overrepresentation analysis disclosed 12 pathways overrepresented in grade III but not in grade II PF-EPs, whose significance needs to be further investigated, i.e., ortocotropin releasing factor receptor signaling pathway, thyrotropin-releasing hormone receptor signaling pathway, nicotine pharmacodynamics pathway, oxytocin receptor mediated signaling pathway, opioid proenkephalin pathway, metabotropic glutamate receptor group II pathway, inflammation mediated by chemokine and cytokine signaling pathway, enkephalin release, beta3 adrenergic receptor signaling pathway, blood coagulation, angiotensin II-stimulated signaling through G proteins and beta-arrestin, and 5HT4 type receptor mediated signaling pathways.

By limiting the investigation to cancer related proteins, differences between grade II and grade III specimens were disclosed. Their pathway overrepresentation analysis compared with non-cancer proteins confirmed the overrepresentation in grade III of blood coagulation and de novo purine and pyrimidine deoxyribonucleotide biosynthesis, while grade II PF-EPs were characterized by proteins involved in regulation of actin cytoskeleton and plateled activation pathways.

In addition, top-down relative label free quantitation revealed higher levels in grade III PF-EPs of alpha and beta hemoglobin subunits and of GFAP fragments 388–432. Recent studies focused the attention on the expression of hemoglobin chains in cells different from the erythrocyte line, indicating in them an interesting correlation with hemorphins and brain tumors [43]. Alteration of hemoglobin levels was observed in breast [44] and anaplastic thyroid [45] tumors. Interestingly, genes involved in gliomas development (IGF2, H19, PHLDA2/TSSC3, TRIM3, SLC22A18) are located in the gene locus 11p15.5 of the beta hemoglobin chain. It is furthermore noteworthy to underline that the previously discussed hemorphin peptides determined in the cerebrospinal fluid of posterior fossa tumors [41] are endogenous fragments of the hemoglobin beta chain, whose production seems to be blocked in the presence of a brain tumor. Therefore, in light of the data present in the literature, the expression of globin chains in ependymoma and the recognition of their higher level in the most aggressive phenotype confirm the need for a thorough investigation to deeply understand the role of hemoglobin and opioid peptides in oncogenesis.

## 4. Materials and Methods

### 4.1. Chemicals

Iodoacetamide (IAA), DL-dithiothreitol (DTT), Tris-HCl, sodium deoxycholate, urea powder, sodium dodecyl sulfate (SDS), Tergitol-type NP-40, and acetone were from Sigma-Aldrich (St. Louis, MO, USA). The 2,2,2-trifluoroacetic acid (TFA) and the sodium chloride were from Mallinckrodt Baker B.V. (Deventer, The Netherlands) and Fluka (Sigma-Aldrich Chemie GmbH, Buchs, Switzerland), respectively. Acetonitrile (ACN), methanol (MeOH), ethanol (EtOH), and formic acid (FA), all of LC-MS grade, and EDTA were purchased from Merck (Darmstadt, Germany). Trypsin (Gold MS Grade) was from Promega (Madison, WI, USA), and Halt™ Protease and Phosphatase Inhibitor Cocktail was from USAThermo Fisher Scientific (Rockford, IL, USA). Dye reagent for total protein content assay was purchased from Bio-Rad Laboratories (Hercules, CA, USA). Bovine serum albumin (BSA) was from Sigma Aldrich (St. Louis, MO, USA).

### 4.2. Instrumentation

Tissue homogenization and sonication were carried out by means of Wheaton^®^ 903475 Overhead Stirrer apparatus (Wheaton, Millville, NJ, USA) and Branson sonifer 450 (Branson Ultrasonics, Danbury, CT, USA), respectively. Total protein concentration was determined in duplicate by Bradford assay (Bio-Rad Laboratories, Hercules, CA, USA) and UV-Vis spectrophotometer (8453 UV-Vis Supplies, Agilent Technologies, Waldbronn, Germany) detector using BSA as the protein of reference. For sample centrifugation, thermostated centrifuge SL16 R (Thermo Fisher Scientific, Langenselbold, Germany) and Mini Spin (Eppendorf AG, Germany) were used as specified for sample treatment. HPLC-ESI-MS/MS analyses were performed on UltiMate 3000 RSLCnano System (Dionex, Sunnyvale, CA, USA) coupled to Orbitrap Elite MS detector with ESI or EASY-Spray nanoESI sources (Thermo Fisher Scientific), as specified elsewhere.

### 4.3. Sample Collection and Treatment

Tumor tissues were obtained from 12 pediatric patients affected by ependimoma brain tumor (7 males, 5 females, 0.7–16 years, mean age 7.9 years) who underwent the surgical removal of the tumor at our institution. The study was realized under the approval of the local Ethical Committee (Prot.N 0034878/16). Table 2 reports all sample data together with grade classification, tumor localization, and primary ependymoma tumor diagnosis. Five ependymoma tissue samples (EP) were from patients affected by grade II ependymoma (EP1–5), while seven samples were from patients affected by grade III ependymoma (EP6–12), according to WHO classification. Within this list, two samples were from supratentorial ependymomas (EP 4 and EP10), while the others were from posterior cranial fossa ependymomas.

Tumor tissues were collected during surgery under sterile conditions and immediately stored at −80 °C. Before pretreatment, tissue samples were thawed on ice, washed with cold phosphate-buffered saline (PBS) solution containing the protease and phosphatase inhibitor cocktail, and weighed. Tissues were then added to a volume of water/ACN (70/30, *v/v*) solution, containing 0.4% TFA (*v/v*) and the protease and phosphatase inhibitor cocktail (1:100, *v/v*), in order to have a final concentration of 0.2 mg/µL (tissue/solution) per sample. Then, they were homogenized for 1 min and thereafter sonicated for 3 × 1 min cycles. Following centrifugation at 23,791× *g* for 30 min at 4 °C, the resulting supernatant, corresponding to the acid-soluble fraction of tissue homogenate, was collected for direct LC-MS top-down proteomic analysis, while the pellet, i.e., the acid-insoluble fraction, underwent enzymatic digestion protocol for shot-gun proteomic analysis.

The pellets were weighted and added to a volume of Radioimmunoprecipitation assay (RIPA) buffer solution [composition: Tris-HCl 50 mM pH 8, NaCl 150 mM, sodium deoxicholate 0.5% (*v/v*), SDS 0.1% (*v/v*), NP-40 1% (*v/v*), EDTA 1 mM] containing the protease inhibitor cocktail (1:100, *v/v*) in order to obtain a final concentration of 20 mg/mL (pellet/RIPA buffer). Pellet dissolution and homogenization were performed by potter mixing at 4 °C per 1 min. Samples were stored at 4 °C for 30 min, vortex mixed in repeated cycles, then sonicated at 4 °C for 2 min × 180 W in 10 sec alternating rests. Samples were then centrifuged at 10,000 *g*/min at 4 °C per 10 min. The supernatant was collected, and the total protein content was measured by Bradford assay using bovine serum albumin as the reference. A supernatant volume was added to 6× volume of EtOH/MeOH/acetone/water (49:24.5:24.5:5:2, *v/v*) cold solution, vortex mixed, and stored overnight at −80 °C to allow protein precipitation. Samples were then centrifuged at 23,791× *g* per 30 min at 4 °C. The supernatant was discarded, and the resulting pellet was washed by adding 1 mL water/acetone solution (20:80, *v/v*), mixed, and—after 30 min storage at −80 °C—centrifuged to recover the pellet. Up to 4 washing cycles were performed following this procedure. The resulting pellet was then evaporated to dryness and suspended in a volume of 100 mM Tris-HCl pH 7.8 6 M urea buffer solution to obtain a final total protein concentration of 2 μg/μL. To facilitate pellet dissolution, the sample underwent 3 × 10 min cycles of ultrasonication. After a further step of centrifugation, the total protein content was determined by Bradford assay to assess the protein digestion protocol. Before digestion, samples were added to 10 mM DTT (final concentration in the sample) and incubated for 1 h at 37 °C for disulfide bonds reduction followed by 20 mM IAA (final concentration in the sample) and incubation for 1 h at 37 °C in the dark for sulfhydryl groups alkylation. The excess IAA was removed by the addition of DTT and incubation at 37 °C for 20 min. Proteins digestion was carried out overnight at 37 °C by addition of trypsin enzyme 1:50 (*w/w*) with respect to the value of total protein content. Enzymatic digestion was stopped by the addition of 0.1% FA (*v/v*, final content in the sample). The resulting peptide mixture underwent to a clean-up step by C18 ZipTip pipette tips (Millipore Corporation; Billerica, MA, USA). Samples were stored at −80 °C until LC-MS analysis.

### 4.4. LC-MS Analyses Operating Conditions

For top-down proteomic analyses, the chromatographic column used was Zorbax 300 SB-C8 (3.5 µm, 1.0 i.d., ×150 mm) (Agilent Tecnologies (Santa Clara, CA, USA) in coupling to Acclaim PepMap300 trap cartridge (Thermo Fisher Scientific) in eluent A with the following step gradient elution using eluent A (FA 0.1%, *v/v*) and solvent B (water/ACN 20:80, *v/v*, 0.1% FA, *v/v*): (i) from 0% to 2% B (2 min), (ii) from 5% to 70% B (38 min), (iii) from 70% to 99% B (5 min), (iv) from 99% to 5% B (2 min), (v) 5% B (5 min) at a flow rate of 50 μL/min. The injection volume was 20 μL, corresponding to 7.8 μg of total protein concentration after opportune sample dilution with 0.1% (*v/v*) FA aqueous solution. Chromatographic separations were performed in triplicate at a thermostated temperature of 40 °C. The Orbitrap Elite instrument was operating in positive ionization mode at a 60,000 resolution in 350–2000 m/z scan filter range in Data-Dependent Scan (DDS) mode and performing MS/MS fragmentation by High-energy collisional dissociation (HCD) of the 5 most intense signals of each full scan MS spectrum. The minimum signal was set to 500.0 and the isolation width to 5.00 m/z. Normalized collision energy was set at 35.0. Capillary temperature was 300 °C, and the source voltage was +4 kV. Acquisition started at 4 min in order to avoid salt source contamination in the first minute of elution.

For bottom-up analyses, the chromatographic column used was EASY-Spray column 15 cm × 50 µm ID, PepMap C18 (2 µm particles, 100 Å pore size) in coupling with Acclaim PepMap100 cartridge (C18, 5 μm, 100 Å, 300 μm i.d. × 5 mm) (Thermo Fisher Scientific) in gradient elution using eluent A (FA 0.1%, *v/v*) and solvent B (ACN:FA 99.9:0.1, *v/v*) and the following steps: (i) 5% B (2 min), (ii) from 5% to 60% B (120 min), (iii) from 60% to 99% B (15 min), (iv) 99% B (10 min), (v) from 99% to 5% B (2 min), (v) 5% B (13 min) at a flow rate of 0.3 μL/min. The injection volume was 5 μL, corresponding to 1.25 μg of total protein concentration after opportune sample dilution with 0.1% (*v/v*) FA aqueous solution. Chromatographic separations were performed in triplicate at a thermostated temperature of 35 °C. The Orbitrap Elite instrument was operating in positive ionization mode at a resolution of 60,000 in 350–2000 m/z scan filter range in Data-Dependent Scan (DDS) mode and performing MS/MS fragmentation by Collision-induced dissociation (CID) of the 20 most intense signals of each full scan MS spectrum. The minimum signal was set to 500.0 and the isolation width to 2.00 m/z. Normalized collision energy was set at 35.0. Capillary temperature was 250 °C, and the source voltage was +1.5 kV. MS/MS spectra acquisition was performed in the linear ion trap at normal scan rate. Acquisition started at 4 min in order to avoid salt source contamination in the first minute of elution. All samples were analyzed in triplicate.

### 4.5. Data Analysis

LC-MS proteomic data were elaborated by the Xcalibur software (version 2.0.7 SP1, Thermo Fisher Scientific) by both manual inspection and Proteome Discoverer 1.4 software (version 1.4.1.14, Thermo Fisher Scientific) elaboration and using ExPASy UniProtKb database and proteomics tools (http://www.expasy.org/tools/) for protein characterization.

Particularly, protein and PTMs identification by top-down analysis was assessed by matching the monoisotopic molecular mass of the protein/peptide, obtained by full scan MS spectra deconvolution, with amino acid sequencing by manual inspection of the tandem MS spectra of the relative m/z multicharged ions and using ExPASy UniProtKb database and proteomics tools for characterization and ProSight Lite v1.4 free software [22] for experimental/theoretical spectra matches, tandem MS spectra annotation, and PTMs localization. Relative quantitation of the protein/peptide was assessed by comparing the peak area values (signal/noise ratio >5) of the eXtracted Ion Current (XIC) plots, obtained by extraction from the total ion current (TIC) profile of the ion current signals of the relative multiple charged ions (m/z). Significant differences in protein quantitation between samples were evaluated by submitting the mean area values of three technical replicates per sample to T-test statistical analysis and considering a *p*-value < 0.05 as statistically significant.

MS and MS/MS data obtained from shot-gun analyses were elaborated by Proteome Discoverer 1.4 software (version 1.4.1.14, Thermo Fisher Scientific) based on SEQUEST HT cluster as the search engine against the Swiss-Prot Homo Sapiens proteome (UniProtKb, Swissprot, homo+sapiens released in March 2018) with the following settings: minimum precursor mass 350 Da; maximum precursor mass 10,000 Da; total intensity threshold 0.0; minimum peak count 1; signal-to-noise (S/N) threshold 1.5; precursor mass tolerance 10 ppm; fragment mass tolerance 0.5 Da, use average precursor mass “False”, use average fragment mass “False”. Trypsin enzyme was set with a maximum of 2 missed cleavage sites. The minimum and the maximum peptide lengths were 6 and 144 residues, respectively. Dynamic methionine oxidation (+15.99 Da) and static carbamidomethylation of cysteine (+57.02 Da) were also set. Protein and peptide spectra matches were validated by the calculation of false discovery rate (FDR) using the Percolator node. The strict target FDR value was set at 0.01, while the relaxed value was set at 0.05. Identification results were further filtered for: high peptide confidence; peptide rank 1; 2 peptides identified per protein; peptide length > 9 amino acids. For top-down data elaboration by Proteome Discoverer, the following result filters were applied: high peptide confidence and peptide rank 1.

Venn diagrams (http://bioinformatics.psb.ugent.be/webtools/Venn/) were used for sample grouping analysis. Gene ontology (GO) analysis and pathways classification and over-representation of the identified proteins were performed by Protein ANalysis THrough Evolutionary Relationships (PANTHER) Classification System (version 11.0) [46] and KEGG mapper [47] tools. Protein–protein association networks were investigated by STRING tool [48]. The cancer related genes list of reference for shotgun data elaboration was reported in the Human Protein Atlas (http://www.proteinatlas.org) [49].

## 5. Conclusions

The present investigation represents, to the best of our knowledge, the first protein fingerprinting of ependymoma tissue obtained by the application of a top-down/bottom-up integrated proteomic platform, thus revealing the first hallmark of its intact proteome, PTMs and naturally occurring bioactive peptidome, and the first preliminary proteomic study extended to ependymoma tissue of different grade and localization.

The results that emerged from this pilot study seem consistent with different protein profiles associated with ependymoma tissues of diverse tumor localizations, i.e., supratentorial and posterior fossa, and WHO histopathological grade in terms of either selective protein levels and distribution or gene ontology classification and pathways overrepresentation of both cancer and non-cancer related proteins. Considering the rarity of pediatric ependymoma, our results, although obtained from a limited number of samples, contribute novel information to the developing mosaic of molecular characteristics of the tumor related to its onset and progression and to its development in different regions of the brain, outlining potential proteins marking tumor location and/or aggressiveness or molecular pathways to be further investigated. It is, however, necessary in future studies to underline the importance of expanding the cohort of samples under study in order to confirm the significance of the results obtained, although with the limits connected with the low incidence of this type of neoplasm.

The low incidence of neoplasia (the difficulty in stratifying risk) linked to an often non-univocal histological picture and the lack of therapeutic alternatives to surgery and radiotherapy make ependymoma one of the most difficult challenges in childhood neurosurgery. The elucidation of the pathogenetic mechanisms of this tumor could provide a key understanding of its clinical, diagnostic, prognostic, and therapeutic implications. In this purpose, proteomics—looking at the gene products and their post translational modifications—adds new hints and complementary information to genomic based studies in the comprehension of the molecular features underlining this still cryptic and unclear tumor, which lacks specific biomarkers and targeted therapies.

## Figures and Tables

**Table 1:** Proteins and peptides identified in ependymoma (EP) tumor tissues following the top-down approach.

Uniprot Accession	Protein Name	Amino Acid Position •	[M+H]^+^ Theory	[M+H]^+^ Experiment	PTMs	*p*-Score *
P69905	Hemoglobin α-Chain	2–142 (Chain)	15117.89	15117.90	-	1.5 × 10^−21^
		2–32	3195.65	3195.66	-	8.8 × 10^−40^
P68871	Hemoglobin β-Chain	2–147 (Chain)	15858.26	15858.25	-	1.1 × 10^−47^
P63313	Thymosin β10	2–44 (Chain)	4934.53	4934.54	Acetylation _N-terminal_	1.2 × 10^−38^
	Thymosin β10-IS ^#^ [18,19]	2–42	4734.41	4734.42	Acetylation _N-terminal_	-
	Thymosin β10-SEIS ^§^	2–40	4518.34	4518.36	Acetylation _N-terminal_	-
P62328	Thymosin β4	2–44 (Chain)	4961.50	4961.50	Acetylation _N-terminal_	2.0 × 10^−30^
	Thymosin β4	2–44 (Chain)	4977.49	4977.49	Acetylation _N-terminal_ Oxidation M_7_	5.0 × 10^−19^
	Thymosin β4 des-ES (C-terminal)	2–42	4745.42	4745.43	Acetylation _N-terminal_	1.2 × 10^−15^
	Thymosin β4 des-AGES (C-terminal)	2–40	4617.36	4617.36	Acetylation _N-terminal_	4.2 × 10^−33^
	Thymosin β4 (2-19)	2–19	2151.10	2151.10	Acetylation _N-terminal_	1.8 × 10^−36^
	Thymosin β4 (2-14)	2–14	1566.70	1566.70	Acetylation _N-terminal_	1.2 × 10^−19^
P20962	Parathymosin	2–102 (Chain)	11435.17	11435.15	Acetylation _N-terminal_	1.9 × 10^−29^
P61604	10 kDa Heat Shock Protein	2–102 (Chain)	10836.85	10836.87	Acetylation _N-terminal_	8.6 × 10^−22^
P59665	α-Defensin 1 ^#^ [20,21]	65–94	3440.52	3440.53	Disulfide bonds _(66__→94, 68__→83, 73__→93)_	-
P59665/6	α-Defensin 2 ^#^ [20,21]	66–94	3369.48	3369.49	Disulfide bonds _(66__→94, 68__→83, 73__→93)_	-
P59666	α-Defensin 3 ^#^ [20,21]	65–94	3484.51	3484.51	Disulfide bonds _(66__→94, 68__→83, 73__→93)_	-
P56385	ATP Synthase Subunit e, Mitochondrial	2–69 (Chain)	7798.29	7798.30	-	3.1 × 10^−15^
P18859	ATP Synthase Coupling Factor 6, Mitochondrial	33–108 (Chain)	8955.55	8955.48	-	2.9 × 10^−26^
P14854	Cytochrome C Oxidase Subunit 6B1	2–86 (Chain)	10093.67	10093.66	Acetylation _N-terminal_	7.1 × 10^−16^
P14136	Glial Fibrillary Acidic Protein	388–432	5206.73	5206.74	-	4.5 × 10^−24^
		388–432	5207.72	5207.74	Deamidation Q_388_ or CitrullinationR_390_	2.4 × 10^−32^
		388–432	5208.70	5208.72	Deamidation Q_388_ or CitrullinationR_390_ Citrullination R_416_	1.8 × 10^−24^
		388–432	5209.68	5209.69	Deamidation Q_388_ or CitrullinationR_390_ Citrullinations R_406,416_	4.6 × 10^−28^
		388–431	5075.69	5075.70	-	1.7 × 10^−32^
			5076.67	5076.69	Citrullination R_390 or 406 or 416_	1.4 × 10^−21^
			5077.66	5077.67	Deamidation Q_388_ or Citrullination R_390_ Citrullination R_416_	4.6 × 10^−29^
		15–36	2185.13	2185.14	-	1.3 × 10^−35^
		388–430	4976.62	4976.61	-	6.2 × 10^−29^
		388–430	4977.61	4977.63	Deamidation Q_388_ or Citrullination R_390_	1.3 × 10^−37^
		388–430	4978.59	4978.60	Deamidation Q_388_ or Citrullination R_390_, Citrullination R_416_	1.3 × 10^−48^
		388–430	4979.57	4979.59	Deamidation Q_388_ or Citrullination R_390_ Citrullinations R_406,__416_	4.4 × 10^−44^
		398–432	4035.11	4035.12	-	3.7 × 10^−35^
		398–432	4036.10	4036.11	Citrullinations R_406_	9.1 × 10^−19^
		398–432	4036.10	4036.10	Citrullinations R_416_	2.2 × 10^−20^
		398–432	4037.08	4037.08	Citrullinations R_406,__416_	2.1 × 10^−26^
		388–405	2028.07	2028.08	-	2.3 × 10^−37^
		388–405	2029.06	2029.06	Deamidation Q_388_ or Citrullination R_390_	4.4 × 10^−36^
		416–432	2028.02	2028.02	-	2.8 × 10^−31^
		22–36	1463.86	1463.86	-	9.4 × 10^−23^
		388–415	3197.73	3197.74	-	6.0 × 10^−21^
		388–415	3198.72	3198.72	Deamidation Q_388_ or Citrullination R_390_	2.1 × 10^−18^
		406–432	3197.68	3197.68	-	1.0 × 10^−30^
		406–432	3198.66	3198.67	Citrullination R_416_	3.3 × 10^−37^
P08670	Vimentin	424–466	4953.53	4953.54	-	1.6 × 10^−48^
		424–466	4954.51	4954.53	Citrullination R_440_ or R_450_ or Deamidation Q_453_	1.2 × 10^−20^
		422–466	5180.66	5180.67	-	5.0 × 10^−25^
		422–466	5181.64	5181.65	Deamidation N_422_ or Citrullination R_424_	6.4 × 10^−40^
		422–466	5182.63	5182.64	Deamidation N_422 or 427_ or Citrullination R_424_ Citrullination R_424 or 450_	6.7 × 10^−27^
		430–466	4225.18	4225.19	-	7.2 × 10^−18^
		430–466	4226.17	4226.18	Citrullination R_450_	2.5 × 10^−23^
		443–466	2777.37	2777.37	-	6.3 × 10^−20^
		443–466	2778.35	2778.37	Deamidation N_456_	2.6 × 10^−21^
		443–466	2778.35	2778.36	Citrullination R_450_ or Deamidation Q_453_	5.6 × 10^−36^
		444–466	2664.29	2664.29	-	7.7 × 10^−25^
		444–466	2665.27	2665.28	Deamidation Q_460_	1.0 × 10^−18^
		444–466	2665.27	2665.27	Citrullination R_450_	2.4 × 10^−30^
P07108	AcylCoA Binding Protein	2–87 (Chain)	9950.00	9950.03	Acetylation _N-terminal_	1.1 × 10^−33^
P07108	AcylCoA Binding Protein natural variant M→V	2–87 (Chain)	9918.03	9918.04	Acetylation _N-terminal_	4.2 × 10^−37^
P04271	S100B	2–92 (Chain)	10618.03	10618.02	Acetylation _N-terminal_	1.9 × 10−39
P01011	α-1-Antichimotrypsin	390–423	4023.18	4023.20	-	2.6 × 10^−35^
		387–423	4352.34	4352.36	-	2.7 × 10^−31^
P01009	α-1-Antitrypsin	384–418	4046.20	4046.21	-	7.1 × 10^−43^
Q16555-2	Dihydropyrimidinase-related protein	521–570	5305.80	5305.81	-	2.9 × 10^−29^
		521–572	5475.91	5475.92	-	1.5 × 10^−33^
B4DV12	Ubiquitin	1–76 (Chain)	8560.63	8560.64	-	6.8 × 10^−43^
	Ubiquitin des-GG (C-terminal)	1–74	8446.58	8446.59	-	8.6 × 10^−49^
P05204	Non-histone chromosomal protein HMG-17	2–90 (Chain)	9258.01	9258.02	Deamidation N_72_	1.5 × 10^−60^
P62805	Histone H4	2–103 (Chain)	11300.39	11300.49	Acetylation _N-terminal_, Dimethylation K_21_	4.0 × 10^−18^
		2–103 (Chain)	11342.40	11342.36	Acetylation _N-terminal_ Acetylation K_17_ or K_32_ Dimethylation K_21_	2.4 × 10^−16^
Q6FI13	Histone H2A type 2-A	2–130 (Chain)	13998.87	13998.91	Acetylation _N-terminal_	1.2 × 10^−27^
Q5QNW6	Histone H2B Type 2-F	2–126 (Chain)	13781.54	13781.57	-	3.1 × 10^−20^
P00441	Superoxide dismutase [Cu-Zn]	2–154 (Chain)	15835.87	15836.00	Acetylation _N-terminal_, Disulfide bond (_58__→147)_	6.5 × 10^−20^

• (Chain) indicates the identification of the entire protein chain; ^#^ Based on previous identifications, relative reference reported; ^§^ Based on previous identification, unpublished data (MS/MS data unavailable); * Resulting from Prosight light [22] MS^2^ experimental/theoretical spectra matching (data in Table S1).

**Table 2:** Ependymoma tumor specimens description.

Specimen ID	Patient Age (Year)	Patient Sex	Tumor Grade	Tumor Site *	Newly Diagnosed (N) Tumor Recurrence (R)
EP3	8	M	WHO II	PF (IV ventricle)	N
EP4	12	M	WHO II	ST (right lateral ventricle)	R
EP5	16	F	WHO II	PF (IV ventricle/cerebellopontine angle)	N
EP6	8	F	WHO III	PF (IV ventricle)	R
EP7	12	M	WHO III	PF (IV ventricle)	N
EP8	13	M	WHO III	PF (IV ventricle)	N
EP9	0.7	M	WHO III	PF (IV ventricle)	N
EP10	6	M	WHO III	ST (frontal/left parietal)	N
EP11	1	F	WHO III	PF (IV ventricle)	N
EP12	1	M	WHO III	PF (IV ventricle)	N

* PF, posterior fossa; ST, supratentorial.

## Supplementary Materials

The following are available online at https://www.mdpi.com/2072-6694/12/3/674/s1, Table S1: Top down identification data of the proteins and peptides listed in Table 1 as resulting from MS/MS theoretical/experimental spectra matches, Figure S1: Relative quantitation of the proteins, identified by top-down approach, exhibiting a *p* value <0.01 statistically significant variation between ST- and PF-EPs, Table S2: Protein identification data per analyzed specimens following MS/MS data elaboration by Proteome Discoverer software, Table S3: (A–E): Uniprot accession list of the protein identified in each analyzed sample by shotgun proteomic analysis (A), Venn diagram grouping of the proteins identified per sample (B), Venn diagram grouping of the proteins identified in grade II PF-EPs (C), Venn diagram grouping of of the proteins identified in grade III PF specimens (D), full list of the pathways classified and overrepresented in grade II and grade III PF-EPs (E).
