# Supplementary material for: Ependymoma Pediatric Brain Tumor Protein Fingerprinting by Integrated Mass Spectrometry Platforms: A Pilot Investigation
Source: Cancers (Basel). 2020 Mar 13;12(3):674. doi: 10.3390/cancers12030674 (PMC7140025; doi:10.3390/cancers12030674)
Supplement: Supplementary file 1 [file cancers-12-00674-s001.zip › Figure S1.pdf]

# Supplementary Materials: Ependymoma Pediatric Brain Tumor Protein Fingerprinting by Integrated Mass Spectrometry Platforms: A Pilot Investigation

**Diana Valeria Rossetti, Luca Massimi, Claudia Martelli, Federica Vincenzoni, Susanna Di Silvestre, Gianluca Scorpio, Gianpiero Tamburrini, Massimo Caldarelli, Andrea Urbani and Claudia Desiderio**

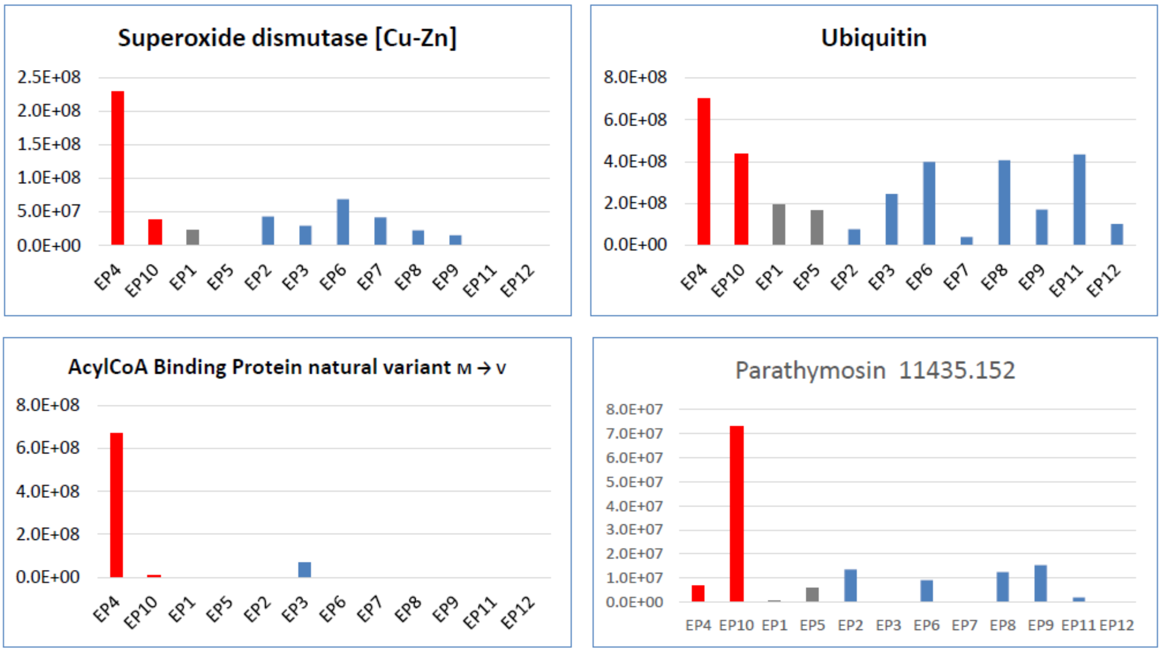


**Figure S1.** Proteins showing statistically significant higher levels in ST with respect to PF ependymomas ($p < 0.05$).

© 2020 by the authors. Licensee MDPI, Basel, Switzerland. This article is an open access article distributed under the terms and conditions of the Creative Commons Attribution (CC BY) license (http://creativecommons.org/licenses/by/4.0/).
